# Neurons in the pigeon caudolateral nidopallium differentiate Pavlovian conditioned stimuli but not their associated reward value in a sign-tracking paradigm

**DOI:** 10.1038/srep35469

**Published:** 2016-10-20

**Authors:** Nils Kasties, Sarah Starosta, Onur Güntürkün, Maik C. Stüttgen

**Affiliations:** 1 Department of Biopsychology, Faculty of Psychology, Ruhr University, 44780 Bochum, Germany; 2 Institute of Pathophysiology, University Medical Center of the Johannes Gutenberg University, 55128 Mainz, Germany; 3 Focus Program Translational Neurosciences, Johannes Gutenberg University, 55128 Mainz, Germany

## Abstract

Animals exploit visual information to identify objects, form stimulus-reward associations, and prepare appropriate behavioral responses. The nidopallium caudolaterale (NCL), an associative region of the avian endbrain, contains neurons exhibiting prominent response modulation during presentation of reward-predicting visual stimuli, but it is unclear whether neural activity represents valuation signals, stimulus properties, or sensorimotor contingencies. To test the hypothesis that NCL neurons represent stimulus value, we subjected pigeons to a Pavlovian sign-tracking paradigm in which visual cues predicted rewards differing in magnitude (large vs. small) and delay to presentation (short vs. long). Subjects’ strength of conditioned responding to visual cues reliably differentiated between predicted reward types and thus indexed valuation. The majority of NCL neurons discriminated between visual cues, with discriminability peaking shortly after stimulus onset and being maintained at lower levels throughout the stimulus presentation period. However, while some cells’ firing rates correlated with reward value, such neurons were not more frequent than expected by chance. Instead, neurons formed discernible clusters which differed in their preferred visual cue. We propose that this activity pattern constitutes a prerequisite for using visual information in more complex situations e.g. requiring value-based choices.

Animals rambling about their environment harness their knowledge about the relationships between different objects to guide their search for food. Such knowledge can be acquired through repeated co-occurrence of a given stimulus (such as the sight of a tree) and treasured food items (such as fruit). Animals tend to approach stimuli that have signaled the availability of reward in the past, which is an indication that stimulus-reward associations have been formed and affect action selection. In the laboratory, a corresponding situation is arranged through Pavlovian conditioning, in which a conditioned stimulus (CS) is repeatedly paired with an appetitive unconditioned stimulus (US) such as food. When the CS is a discrete visual stimulus, animals will approach it, a phenomenon called ‘sign-tracking’^1^. During the development of a sign-tracking response, the CS is endowed with incentive salience and acquires rewarding properties itself 2. In rats, successful establishment of sign-tracking depends on the intactness of orbitofrontal cortex (OFC), suggesting that OFC neurons subserve the formation of stimulus-reward associations in Pavlovian learning^3^. Indeed, many neurons in macaque OFC fire for reward-predicting visual stimuli, and firing rates scale with the animal’s valuation of the stimulus-associated reward^4^,^5^. Moreover, neural coding of stimulus value has also been found in cortical areas other than OFC, including dorsolateral prefrontal cortex and the lateral intraparietal area^6^,^7^,^8^.

In birds, functions that are implemented by mammalian prefrontal cortex (PFC) are believed to reside in the nidopallium caudolaterale (NCL)^9^. The NCL is an associative endbrain structure whose inbound and outbound connections closely resemble those of the PFC^10^. Similar to PFC, lesions of the pigeon NCL result in deficits in delayed alternation performance^11^, response selection^12^, and reversal learning^13^, while sparing sensorimotor functions^14^. The NCL can to some extent be subdivided based on hodological and histochemical evidence^10^,^15^, but it is unknown whether those subdivisions support different types of functions, as has been suggested for the PFC^16^.

Several single-neuron recording studies have demonstrated that NCL neurons’ firing rates are strongly modulated during the presentation of reward-predicting visual stimuli^17^,^18^,^19^,^20^,^21^, during a post-stimulus delay phase preceding reward delivery^22^,^23^, and during the consumption of food and water rewards^17^,^18^,^19^,^20^,^24^. Possibly, activity in the delay (i.e. post-choice) phase represents a mixture of different processes like information about the relevant visual stimulus^25^, task-related rules^26^, reward expectancy^22^, and information about the upcoming behavioral choice^23^,^27^,^28^. The situation is less explored regarding initial (i.e. pre-choice) stimulus presentation. In principle, modulation during this phase could result from three different sources: visual characteristics of the stimuli (low-level properties such as color or luminance), sensorimotor correlates of stimulus-directed behavior (pigeons emit pecking responses towards reward-predicting visual stimuli), or some learned functional aspects of the stimuli, such as their association with reward. Previous studies have hinted at the possibility that NCL neurons represent the reward value of conditioned visual stimuli^17^,^19^,^20^, based on the observed reward-related neural modulation during post-choice, pre-reward delay phases as well as during reward consumption itself^20^,^22^,^29^. Also, a recent study demonstrated that some NCL neurons either significantly increase or decrease firing for different conditioned stimuli signaling different reward amounts^19^. Here, we asked whether NCL neurons signal the ‘integrated value’ of visual cues, i.e. subjective value integrated across two different dimensions of reward – magnitude and delay to presentation. Moreover, we aimed to factor out sensorimotor contingencies confounded with cue value as a modulator of stimulus-related response modulation^20^. To this end, we subjected birds to a sign-tracking paradigm in which distinct visual cues predicted different outcomes, namely rewards of large or small magnitude, available after a short or long delay, or non-reward. Because the animals’ pecking rate at the visual stimuli scales monotonically with the desirability of the predicted reward, we used this measure to index subjects’ CS valuation^30^.

## Results

### Pecking rate but not pecking force reliably indexes stimulus value

**Figure 1:**
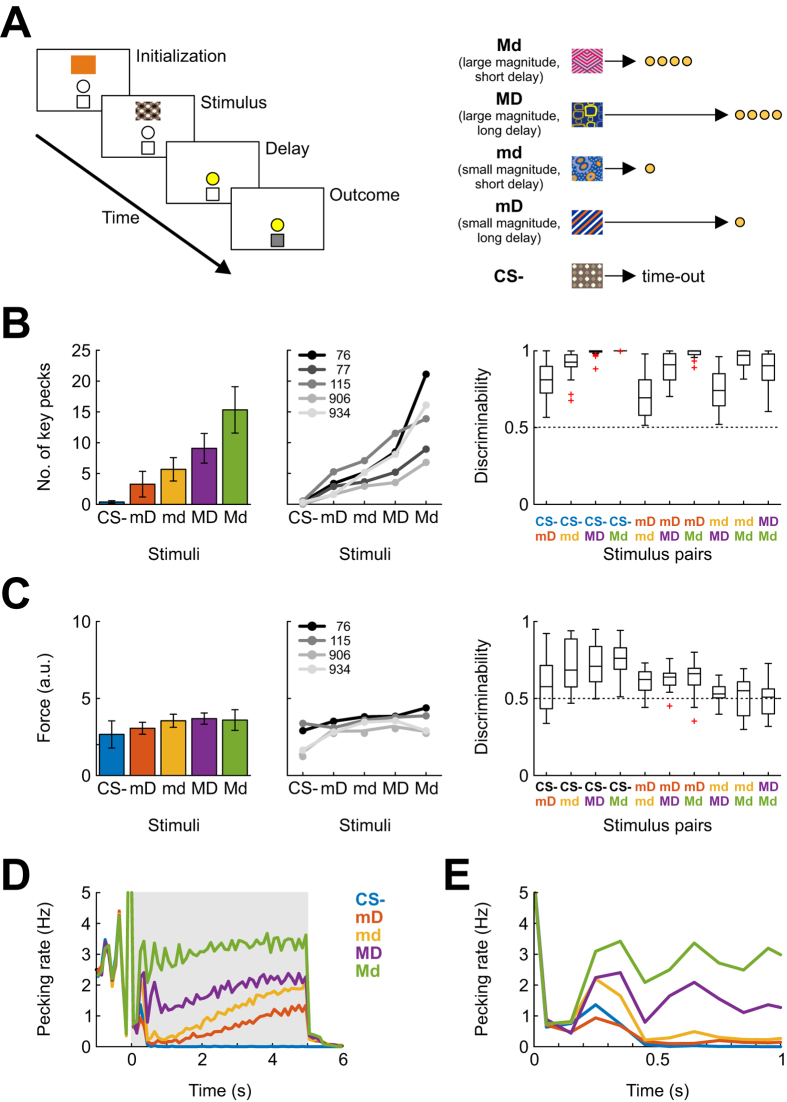
Experimental paradigm and behavioral results. (**A**) Left: Trial outline. Following an intertrial-interval, the initialization stimulus was presented for at least 2 s, after which the first registered key peck initiated the trial. One of five stimuli was then presented for 5 s, during which no behavioral response was required. The sample was then extinguished, followed by either 1) a variable delay (short or long), and a variable reward period during which food was accessible via a food hopper for a short or long period of time, or 2) a 2 s time-out punishment period. Right: Stimuli and their associated reward properties. Stimuli signaled small (“m”) or large (“M”) magnitude of an upcoming reward (duration of food hopper activation), and a small (“d”) or large (“D”) delay until the reward delivery. The most rewarding stimulus Md thus predicted a large reward after a short delay. Rewards were delivered with 50% probability; in case of a reward omission, the delay was increased by the designated feeding time. Brown circles represent grain. Pictorial stimuli shown are similar to the ones used. (**B**) Left: Mean frequency of responses emitted at the conditioned stimuli across all sessions and subjects (mean +/− SD). Middle: Means for individual subjects (across all sessions) Right: Pairwise stimulus discriminabilities computed as the area under the ROC curve for response frequencies across all subjects and recording sessions. (**C**) As in B, but using force of pecking responses rather than frequency. (**D**) Pecking rate during the stimulus presentation phase, averaged across all animals and sessions and plotted in non-overlapping 100-ms bins, shown separately for each visual stimulus. (**E**) As in (**D**), but showing only data for the first second following stimulus onset.

We trained five pigeons on a sign-tracking paradigm in which discrete visual stimuli predicted food rewards of small or large magnitude (“m” and “M”, respectively) and featuring either short or long delays until delivery (“d” and “D”) or the unavailability of food on that trial (CS-; Fig. 1A). Reward magnitude was operationalized as the duration of food availability after stimulus offset and equaled 1–1.5 s (m) or 5–6 s (M), while delay to reward was either 1–2 s (d) or 5–6 s (D; durations were custom-tailored for each bird to achieve discriminative response behavior). For behavioral analysis, we registered all pecking responses directed towards the stimulus during the 5-s sample phase. Subjects were trained until responses clearly and stably differentiated between all stimuli and maintained the same ordinal ranking across at least four of five consecutive sessions. The birds took a median number of 29 sessions to achieve this criterion (range 23–31) and subsequently received movable multi-electrode array implants into the NCL for electrophysiological recordings.

In the vast majority of recording sessions, subjects ranked the stimuli in the same order, namely CS-, mD (small magnitude, long delay), md (small magnitude, short delay), MD (large magnitude, long delay) and Md (large magnitude, short delay). Occasionally, the rank order contained a single inversion. Seven out of forty recording sessions were excluded from analysis either because of stimulus inversion or because one of the four reward-associated stimuli received less than 25 key pecking responses, precluding analysis of neural responses relative to key peck events for that stimulus (see below). In the remaining 33 sessions, response rates reliably indicated stimulus ranking (Friedman test, p < 0.001, Fig. 1B, left and middle panels).

The degree to which subjects’ pecking rates differentiated between stimuli was quantified using the area under the receiver operating characteristic curve (AUROC^31^). The average discriminability value across all sessions and stimulus pairs equaled 0.87 (chance: 0.5, perfect discriminability: 1). The lowest values were obtained for the md-MD stimulus pair (mean: 0.71; Fig. 1B, right panel).

Previous work showed that pecking rate indexes subjective value and predicts choice behavior in subsequent tests of stimulus preference^30^. Having established that cue value strongly influences the rate of responding, we investigated whether it similarly modulates the intensity (i.e. force) of individual key-pecking responses as well. Figure 1C illustrates the mean force of pecking responses directed at a given stimulus, measured with a mechanoelectric transducer attached to the response key. Visual inspection suggests mean force to be largely similar for responses directed at the four reward-predicting stimuli (left and middle panels). Although there was a significant effect of stimulus across all five stimuli (Friedman test, p < 0.001), discriminability values were considerably lower than for response rate (right panel; average discriminability across all sessions and stimulus pairs 0.62; all averages ≤0.75). Importantly, response force did not increase monotonically with stimulus value for three of the four subjects for which force measurements were conducted. Thus, while frequency of conditioned responding was found to be a useful indicator of cue value, force was not.

### Behavioral evidence of stimulus discrimination unfolds within the first half second of stimulus presentation

The above analyses focused on data collapsed across the five seconds of stimulus presentation. In order to visualize the temporal dynamics of conditioned responding, we conducted a time-resolved analysis of key pecking. Figure 1D shows pecking rate as a function of time during the sample phase, separately for each stimulus and averaged over all animals and sessions. Following the peak at time 0 when the animal triggers stimulus presentation, the trajectories of the curves begin to diverge after a few hundred milliseconds. After one second of stimulus presentation, animals did not respond to the CS- (blue) anymore and exhibited maximum pecking rates to the most highly valued stimulus Md (green). For the other three conditioned stimuli, pecking rates increased from low to moderate values within the remaining 4 s of stimulus presentation, as is frequently found for fixed-interval schedules of reinforcement.

Figure 1E provides a close-up of pecking rates in the first second following stimulus onset. It is evident that differential key pecking is present already after 200–300 ms, implying that animals have identified the conditioned stimuli at that time. Accordingly, neural correlates of stimulus valuation are expected to be found as early as 100–200 ms into the sample phase. Note that the drop in pecking rate in the first 200 ms is not the result of a sudden behavioral arrest resulting from stimulus presentation, but simply a reflection of the fact that stimulus presentation was triggered by a key peck (at time 0) and that consecutive pecks are on average separated by about 300 ms. Therefore, the stimulus-dependent reduction of pecking rate compared to the initialization phase (Fig. 1D) indicates behavioral discrimination.

### Neural stimulus discriminability emerges within 200 ms after stimulus onset

**Figure 2:**
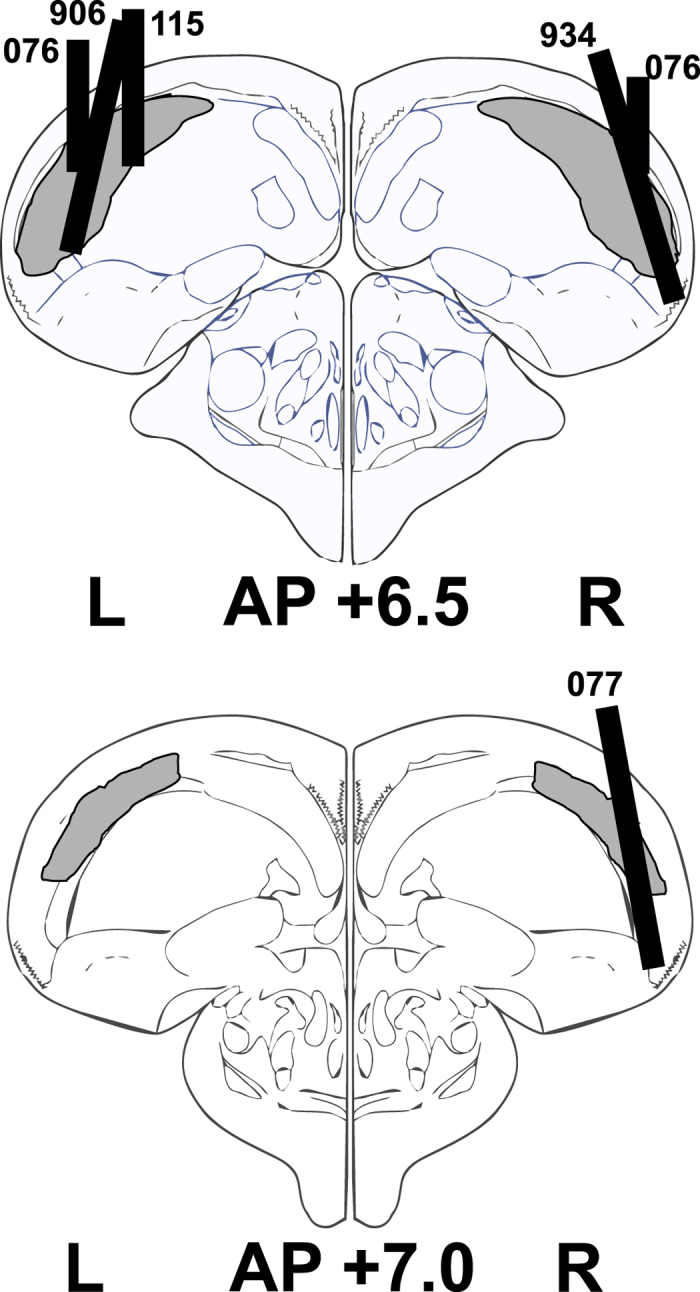
Histological reconstruction of electrode tracks. Sketch shows a coronal section through the pigeon brain at anterior-posterior level of +6.5 mm. Gray shading indicates the NCL as delineated by Herold *et al.*^15^. Black bars indicate electrode tracks, numbers indicate subjects. Brain drawings were performed using CorelDRAW Graphics Suite X5 (http://www.coreldraw.com) and are based on Karten, Harvey J, and William Hodos, “A Stereotaxic Atlas of the Brain of the Pigeon (Columba Livia)”, pp. 82, 86, © 1967 The Johns Hopkins Press^60^. Adapted and reprinted with permission of Johns Hopkins University Press.

**Figure 3:**
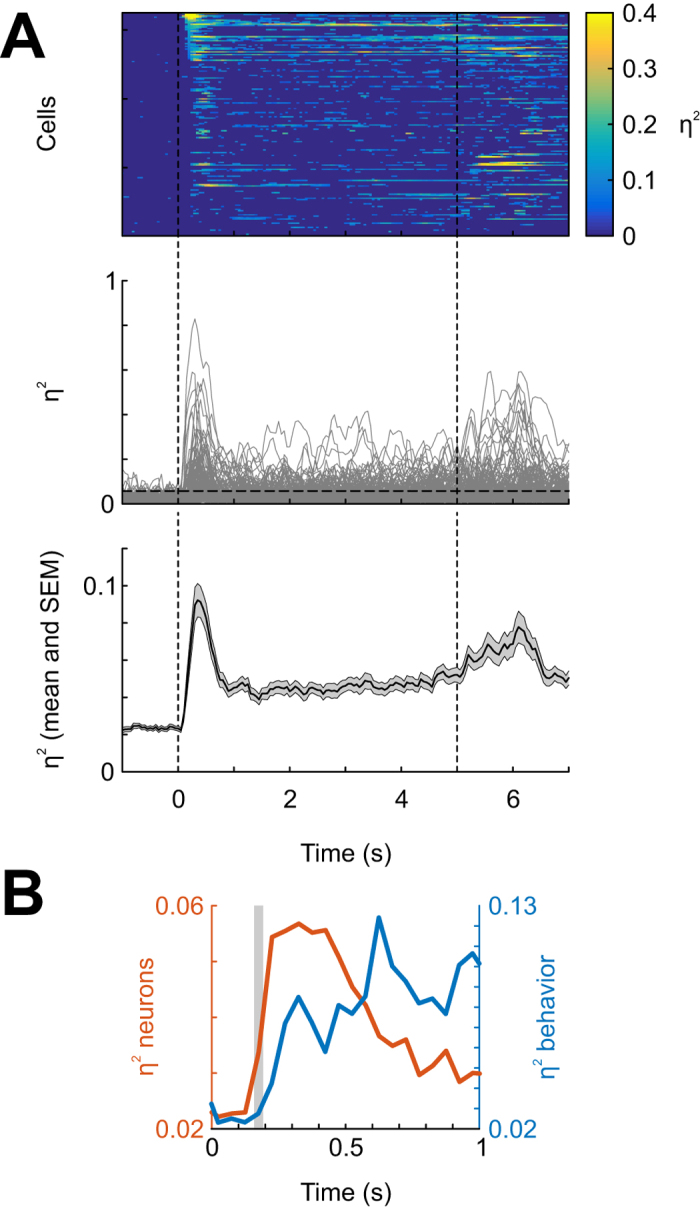
Neural stimulus discriminability during the sample phase. (**A**) Top panel: color-coded stimulus discriminability (η^2^) for each of 162 single neurons recorded in the NCL. η^2^ was computed in 200-ms windows advanced in steps of 50 ms. All bins in which the p-value from a Kruskal-Wallis test on spike count distributions across the visual stimuli was larger than 0.01 were set to 0, and the colorbar was truncated at η^2^ = 0.4. Neurons were sorted by η^2^ values during 100–300 ms. Middle panel: Same data as in the top panel, but each neuron’s η^2^ values are depicted as lines. Horizontal dotted line represents the upper 99^th^ percentile of η^2^ values in the very first 200-ms bin during the 1-s pre-stimulus baseline (0.058). η^2^ values above this line can be considered “statistically significant” at the 1% level. Bottom panel: η^2^ values averaged across all 162 neurons (gray shading: standard error of the mean). Vertical dotted lines reaching across all three panels demarcate stimulus onset and offset. (**B**) Stimulus discriminability (η^2^, computed in non-overlapping 50-ms bins) during the first second of stimulus presentation, shown separately for behavior (pecking rates) and neurons. Gray shading highlights 50-ms bin in which neural but not behavioral discriminability begins to increase.

We analyzed response patterns of 162 neurons from 33 recording sessions. Figure 2 shows the histological reconstruction of recording tracks, which were all found to be within the borders of the NCL^10^,^15^. To gain a first impression of the extent to which NCL neurons discriminate between conditioned stimuli at different time points during the sample phase, we calculated an effect size index (η^2^) for each neuron in 200-ms sliding windows (advanced in steps of 50 ms). This index denotes the fraction of the total variance in firing rates that can be attributed to the factor ‘stimulus’ and thus quantifies the degree to which neural firing rates differentiate between the conditioned stimuli. Figure 3A shows the magnitude of η^2^ as a function of time during the sample phase, individually for all neurons (top and middle panels) and averaged across all 162 neurons (bottom panel). η^2^ increases markedly shortly after stimulus onset, then decreases just as sharply within the first second and stays elevated over baseline levels during the remainder of the sample phase and into the delay preceding the outcome phase. Thus, the neural population differentiates best between the stimuli early in the sample phase (starting around 150–200 ms), leading the first behavioral indication of stimulus discrimination by about 50 ms (Fig. 3B).

### NCL neurons exhibit highly heterogeneous response profiles during stimulus presentation

**Figure 4:**
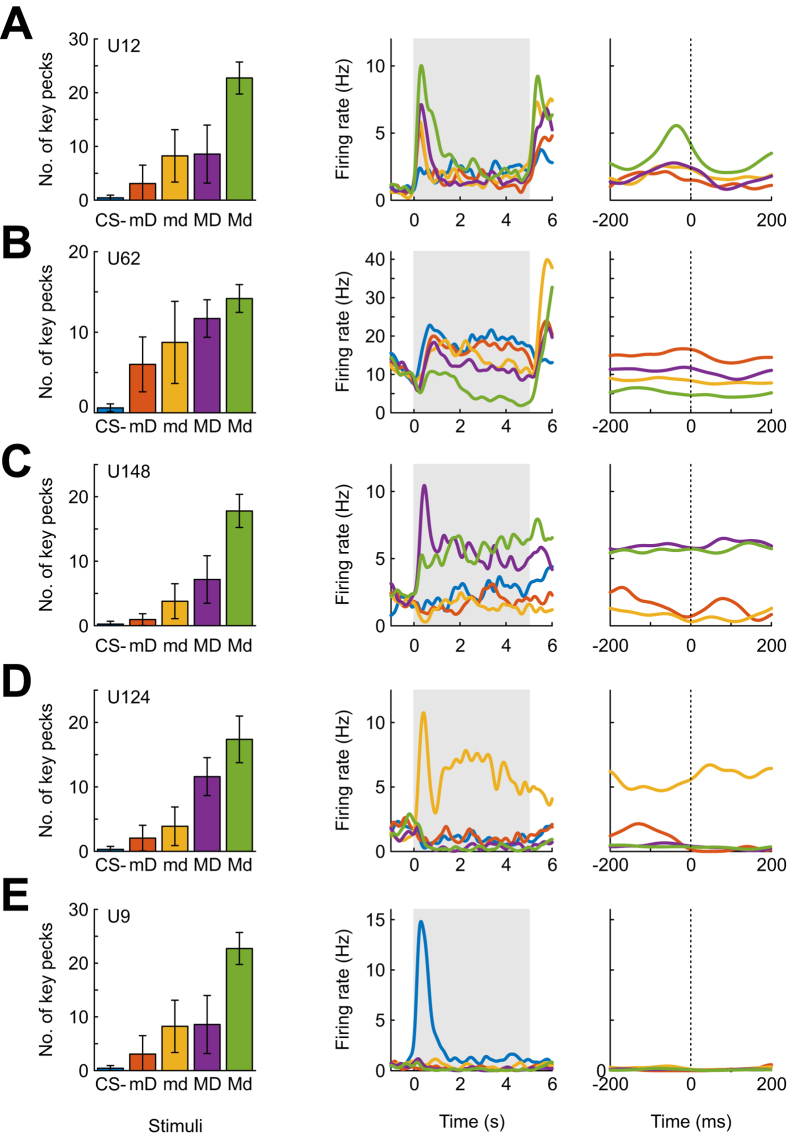
Example NCL neurons. Panels (**A–E**) show data from five different example units. Left panels depict mean response frequency per stimulus for the respective recording session. Middle panels show spike-density functions during presentation of conditioned stimuli, aligned to stimulus onset. Gray area denotes period of stimulus presentation. Right panels: Peri-peck time histogram (PPTH) of neuronal activity centered on individual pecking responses, shown separately for different stimuli. In all panels, color code consistently identifies conditioned stimuli with the same reward quality.

Figure 4 shows data from five example cells which scored among the upper twenty percent of η^2^ values during the early phase of stimulus presentation (100–300 ms). Figure 4A illustrates a neuron which showed an overall increase of firing rate during stimulus presentation (middle panel); in addition, firing rate differed significantly between stimuli (Kruskal-Wallis, p < 0.001), with maximum firing rates to most highly valued stimulus (Md) and minimum firing rates to the least valued stimulus (CS-), a response pattern suggestive of integrated value coding. However, it is not straightforward to assess the source of these activity differences from this kind of analysis, since neural activity in the NCL could also be modulated by the animals’ behavior – recall that birds direct a substantially higher number of key pecks to high-valued stimuli than to low-valued stimuli.

In order to disentangle stimulus- and sensorimotor-related activity modulation, we constructed ‘peri-peck time histograms’ (PPTHs), referencing neural activity to the occurrence of individual key pecks, separately for each conditioned stimulus (the CS- received too few key pecks to allow construction of PPTHs). That way, the frequency of key pecks during presentation of different stimuli is effectively eliminated as a contributing factor to firing rate modulation. For the present example neuron, activity still differed between stimulus conditions after compensating for pecking rate (Kruskal-Wallis, p < 0.001; Fig. 4A, rightmost panel). Moreover, average firing rates were lowest for the least attractive reward-associated stimulus mD and highest for the most attractive stimulus Md, with the rank order of mean firing rates in the time window +/− 100 ms relative to key pecks similar to the rank order of the stimuli, as quantified by Kendall’s rank correlation coefficient (tau = 0.67). Thus, the observed response pattern of this neuron is consistent with the hypothesis that NCL neural activity during stimulus presentation reflects subjective value integrated across two dimensions of reward.

In contrast, Fig. 4B shows an example neuron which fired least for the highest-value and most for the lowest-value stimulus, but again the rank order of activity did not align perfectly with the behavioral responses (tau = −0.67).

Figure 4C shows an example neuron that responds preferentially to two of the stimuli, namely those that predict large reward magnitudes (MD and Md); again, this preference was evident in both stimulus- and peck-referenced analyses (Kruskal-Wallis, p’s < 0.001). This pattern resembles that of neurons responsive to single dimensions of reward which have been described in primate prefrontal cortex^32^. The neuron shown in Fig. 4D exhibited an obvious preference for one of the visual stimuli, and this preference was equally strong in both stimulus- and peck-referenced displays (Kruskal-Wallis, p’s < 0.001). Finally, the neuron in Fig. 4E fired phasically after onset of the CS- but was virtually silent both before and during stimulus presentation for all other stimuli.

Overall, 127/162 units (78%) exhibited significant stimulus-modulated activity during the 5-s sample phase (p < 0.05, Kruskal-Wallis). The vast majority of NCL neurons (109/162, 67%) retained significant stimulus-related activity modulation when neural activity was referenced to key pecks, and the examples shown in Fig. 4 illustrate that some neurons’ response patterns are consistent with a value-coding account, even after compensating for sensorimotor contingencies (i.e., neurons exhibiting significant modulations of their firing rate, along with highly positive or negative tau correlation values).

### No evidence for value coding at the neural population level

**Figure 5:**
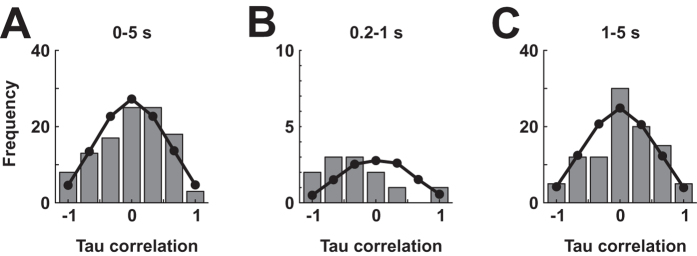
Population analysis. (**A**) Distribution of tau correlation coefficients between behavioral and neuronal responses for all significantly stimulus-modulated neurons (gray bars), computed over the entire duration of the stimulus presentation phase. The black line represents frequencies expected by chance, as obtained from computer simulations. (**B**) As in (**A**), but computed from spikes and key pecks registered between 0.1-1 s after stimulus onset. (**C**) As in (**A**), but using data only from 1-5 seconds during stimulus presentation.

Overall, 29/109 neurons (27%) exhibited perfect tau correlations (+1 or −1) between the animals’ preference and firing rate. However, many more neurons displayed significant firing rate modulations during stimulus presentation that were unrelated to integrated cue value (e.g. Fig. 4D,E). Before concluding that the 29 neurons with perfect tau correlations indeed code for value, it is important to relate their frequency to that expected under the null hypothesis that NCL neurons represented other aspects of the visual stimuli. If a subset of NCL neurons indeed represented cue value, this should lead to an increased occurrence of units with high tau correlations (positive or negative) between behavioral and neuronal response rates. Focusing on all neurons whose firing rates differed significantly between stimuli (Kruskal-Wallis p < 0.05), we statistically compared the empirical distribution of tau values (histogram in Fig. 5A) to that expected by chance (Fig. 5A, black line, obtained from 1,000 simulations in which spike counts were randomly allocated to stimuli) using the chi-square goodness-of-fit test. This account of chance expectancy corresponds to the hypothesis that neurons do discriminate between stimuli (as evidenced by significant response modulation) but are insensitive to their associated reward value.

The empirical distribution of tau values (computed from PPTHs constructed using all pecks in the 5-s sample phase) shown in Fig. 5A is unimodal and centered close to zero; its shape bears close similarity to that of a random distribution, and these distributions accordingly do not differ significantly (chi-square test, p = 0.61). However, the neural response pattern shown in Fig. 3 - stimulus discriminability rising after 200 ms, peaking around 350 ms, declining until about 1000 ms and being maintained at a constant level following stimulus onset - prompted us to repeat the analysis, separately for 200–1000 ms and 1000–5000 ms of the stimulus presentation phase. We obtained no significant difference of observed tau values for either early or late stimulus presentation epochs (chi-square test, p = 0.111 and p = 0.23, and Fig. 5B,C, respectively), although it should be noted that only 29 neurons met our inclusion criteria for the early response epoch, with 12 of these being significantly modulated.

Our analyses so far have focused on single-neuron correlates of integrated value, testing the hypothesis that single neurons’ firing rates scale monotonically with value. A different way of representing integrated value would be to have a larger number of neurons responding to high-value than to low-value stimuli. However, the distribution of preferred stimuli across all neurons was flat and did not deviate significantly from a uniform distribution (chi-square test, p = 0.705, based on 99 units with significantly modulated PPTHs during the late stimulus epoch; there were too few neurons to analyze the early epoch).

The above analyses failed to yield evidence that NCL neurons represent cue value integrated over both dimensions of reward that were manipulated in this experiment. However, it is possible that value coding is indeed present in the NCL, but that individual neurons are sensitive only to a single reward dimension, such as magnitude. Such neurons have been reported in the primate frontal lobe^6^, and we indeed found neurons whose response patterns appeared consistent with this hypothesis (see example in Fig. 4C). Accordingly, we tested whether average firing rates in PPTHs were modulated by the predicted rewards’ magnitude, delay, or both, by means of a two-way analysis of variance (using the full 5-s stimulus epoch). Neurons were classified as ‘pure’ encoders of a certain dimension when they exhibited a significant main effect for that dimension in the absence of both a significant main effect for the other dimension and a significant interaction.

By that criterion, 9/109 (8%) neurons were deemed pure magnitude encoders (including the neuron shown in Fig. 4C), and 16/109 (15%) neurons were deemed pure delay encoders. But again, caution is warranted before relating these numbers to chance expectancy. To get an idea on how many neurons one should expect on the basis of chance, we ran a simulation in which the allocation of spike count distributions to cue values were shuffled. These stimulations showed that, on average, 11–12 pure encoders of each dimension are to be expected, with a 95% range of 6–17. Therefore, this analysis does not provide any evidence for the existence of neurons in the NCL which are sensitive to single reward dimensions.

### NCL neurons cluster according to their preferred visual cue

**Figure 6:**
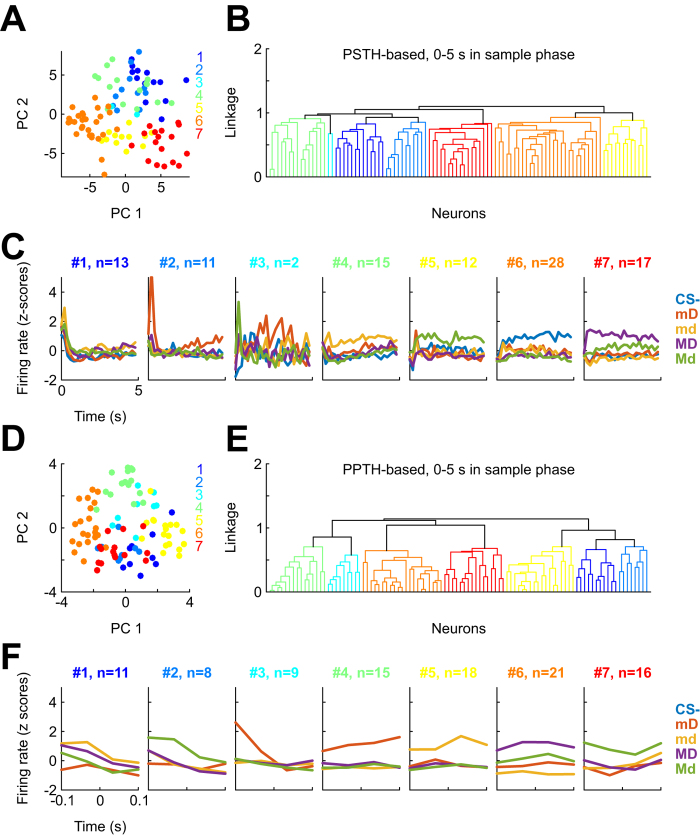
Cluster analysis. (**A**) Scores of the first two principal components for each of 98 neurons showing stimulus discriminability η^2^ > 0.1, obtained after z-transforming five concatenated 100-ms PSTHs (one for each stimulus). Color-coding corresponds to the clusters depicted in (**B**). (**B**) Dendrogram showing linkage between the neurons’ response patterns to the stimuli. Color code as in panel A. (**C**) Average PSTHs for each stimulus (color-coded) for all neurons in each of the seven clusters shown in (**B**). Panel titles indicate cluster ID and the number of neurons in the cluster. (**D,E,F**) Same as (**A,B,C**), but using PPTHs (4 bins of 50 ms, ±100 ms relative to key pecks) as the basis for clustering.

These results provide no evidence for the hypothesis that NCL neural activity during stimulus reflects cue value in our paradigm. However, the fact remains that the vast majority of neurons did show significant stimulus-related modulation both with and without factoring out sensorimotor contingencies. If not their associated reward value, what aspects of the stimuli might be represented by these neurons? Finding an answer to this question is complicated by the strong heterogeneity of NCL neurons’ response patterns. As illustrated by the examples in Fig. 4, some neurons exhibited a graded response to the visual cues, others clearly preferred a single or two stimuli. Accordingly, visual inspection of all neurons’ peri-stimulus time histograms (PSTHs) did not reveal obvious clusters that might aid in the interpretation of NCL response patterns. To conduct an unbiased and systematic investigation into the question whether NCL neurons can be grouped into distinct functional classes, we performed a cluster analysis for 98/162 neurons with moderate to high stimulus discriminability values (η^2^ > 0.1 in any 100-ms bin during the 5-s sample phase). For each neuron, we computed PSTHs (100-ms non-overlapping bins) across the sample phase for all five stimuli, concatenated the five resulting PSTHs, and performed hierarchical cluster analysis with Pearson correlation as similarity measure. Plotting neurons in principal component space (Fig. 6A), however, did not reveal clearly discernible clusters. Hierarchical clustering analysis confirmed this impression, yielding a rather flat dendrogram with relatively small distances between clusters (Fig. 6B). Nonetheless, examination of various cluster sizes ranging from 5 to 16 consistently resulted in the formation of clusters with a clear preference for one of the sample stimuli (Fig. 6C shows average z-transformed PSTHs for a 7-cluster solution). Importantly, a similar result was obtained when using concatenated PPTHs (4 consecutive 50-ms bins within ±100 ms relative to key pecks) rather than PSTHs (Fig. 6D–F), and the clusters were better separated, as visible in the dendrogram in Fig. 6E. Together, these results suggest that the main variable by which different neurons’ response patterns can be separated is stimulus preference, and this becomes more clear when sensorimotor contingencies are factored out by using PPTHs (compare Fig. 6B–E).

## Discussion

We set out to test the hypothesis that stimulus-related modulation of NCL neural activity represents cue value. We subjected pigeons to a Pavlovian sign-tracking paradigm in which different conditioned stimuli predicted different rewards. We then used pecking rates of the animals during stimulus presentation as a behavioral indicator of the subjects’ differential valuation of the conditioned stimuli. We observed stimulus-related activity modulation in the majority of NCL neurons both with and without compensating for sensorimotor contingencies. While some neurons’ firing rates correlated with stimulus valuation, these neurons occurred about as often as expected by chance. Moreover, the numbers of neurons preferring either highly or lowly valued stimuli were roughly equal. Therefore, NCL neural responses are unlikely to reflect integrated cue value, at least under our experimental conditions. However, the strong stimulus-related firing rate modulation we observed, as well as the finding that many neurons preferentially fired for one of the visual cues, matches with the recent description of neurons in corvid NCL that are involved in the representation and maintenance of visual stimulus information for cue-guided behavior. We will first briefly discuss the behavioral findings before moving on to an interpretation of the neurophysiological results.

Two behavioral parameters - frequency and force of pecking responses - were analyzed as possible indices of cue value. Previous work has demonstrated that response frequency scales with reward expectancy and predicts stimulus preference in subsequent forced-choice tests^30^. We are unaware of previous studies investigating whether the force of key pecking is systematically related to stimulus valuation; however, pecking force has been shown to differ between first- and second-order conditioned stimuli, between stimuli predicting food and water reward, and between different degrees of food and water deprivation^33^,^34^. Although we did detect significant differences in response force between differently valued stimuli, these differences were minor in comparison to those obtained with response frequency, especially for the four stimuli associated with reward. Moreover, response force did not increase monotonically with cue value for three of four subjects. It has been suggested that stimulus-directed pecking responses are in fact ‘substituted’ pecks for food consumption^35^, indicated by several stereotypical features such as a closing of the eyes immediately before the forward thrust of the head^36^; this reported stereotypy is consistent with the small variability in pecking force which we observed.

Our finding that response frequency but not response force strongly covaries with cue value shows that the former but not necessarily the latter factor needs to be taken into account when trying to link neural activity and cue value. In freely moving pigeons, many NCL neurons exhibit motor-related firing rate modulation^20^,^21^. Analyzing NCL firing rates relative to key pecks directed at the conditioned stimuli effectively factors out response frequency as a contributor to firing rate modulations, thus providing the opportunity to correlate response frequency (as an indicator of cue value) with neural firing rates largely untainted by sensorimotor contingencies of differential key pecking.

Several previous studies have reported stimulus-related neural response modulation in the NCL. In a recent study from our lab^20^, we recorded NCL neurons from freely moving pigeons in a go/no-go task in which several visual stimuli varying along a single dimension - spatial frequency - were presented, but only a single stimulus was associated with reward. However, because stimuli were arranged to be perceptually highly similar, animals responded to several stimuli, and response rate varied as a function of perceptual distance to the go-stimulus. Many neurons exhibited stimulus-related response modulation, and in a substantial number of cases pecking frequency and neural activity were (mostly negatively) correlated. We did not systematically manipulate reward value in that study, and due to relatively low numbers of key pecks, we were unable to conduct analyses of firing rates referenced to key pecks as in Figs 4, 5, 6 of the present report, thus leaving open the question whether stimulus-related response modulation of these neurons reflected reward value.

A recent study from Koenen and coworkers^19^ asked whether NCL neurons are modulated by reward amount. In a ‘no-choice condition’, pigeons were confronted with either of three differently colored reward-predicting stimuli on each trial. These stimuli were associated with either no, a small, or a large amount of food, and animals were trained to simply peck at each of these stimuli to obtain the associated reward. In the ‘choice condition’, animals instead had to choose either of two simultaneously presented visual stimuli signaling different reward amounts (stimuli and associated reward amounts were identical in both conditions). Based on results from monkey premotor cortex obtained in a similar paradigm^37^, the authors expected to find neural signatures of reward amount in the choice but not the no-choice condition. Contrary to expectation, reward-modulated activity was observed in both conditions.

At first glance, this finding seems to be at odds with our results. A possible explanation of this discrepancy could lie in differing behavioral procedures. Pigeons in that study were operantly conditioned, while we employed sign-tracking, an instance of Pavlovian conditioning. Although our pigeons quickly learned that the different cues were associated with different outcomes and indicated stimulus discriminability by their behavior, they were never required to decide between differently valued stimuli. It is possible that value coding in the NCL is restricted to situations in which value-based choices have to be made. This does not explain why Koenen *et al.* found reward modulation in both the no-choice and the choice condition, but possibly learning to conduct value-based choices between stimuli may alter the neural network to engage NCL neurons to conduct value-based comparisons, and in consequence the network might process the stimuli differently even when the stimuli are presented in a situation not requiring an active decision. If true, our failure to demonstrate value coding in the NCL might be a result of using a simple Pavlovian conditioning paradigm which does not require animals to compute the value of different choice options to guide their forthcoming actions (even though the consistent behavioral differentiation of stimuli clearly implicates a value comparison must occur somewhere). On the other hand, neural representations of value have been found in associative brain areas also in no-choice tasks, for example during trace conditioning in orbitofrontal cortex^38^, a cued-saccade task in the lateral intraparietal area^39^, and a simple go-task in dorsolateral prefrontal cortex^40^, with the caveat that in the latter two studies some kind of active response of the animals was required. Future NCL recording studies using paradigms requiring value-based decisions instructed by visual cues may resolve this question.

What could be the origin of stimulus-related modulation of NCL neural activity that we observed here? Cue-associated reward value seems to be a rather unlikely candidate under the present experimental conditions. Given that the only obvious reason for differential responding was stimulus identity, and that neurons clustered according to their stimulus preference but not any other obvious response characteristic (Fig. 6), basic sensory properties of the visual stimuli could be responsible.

As outlined above, decades of research have fostered the functional equivalence of NCL and PFC (see ref. 41 for review). PFC neurons respond to exteroceptive stimuli and differentiate the physical characteristics of these stimuli to some extent (e.g. dorsolateral PFC^40^ and OFC^42^). Interestingly, during learning of a visual discrimination task the number of cue-responsive PFC neurons increases with task acquisition; after learning, the vast majority of PFC neurons represents not only stimulus characteristics, but also their behavioral significance, such as their reward value or coupling to specific actions^43^.

Just like PFC, the NCL is best characterized as a multimodal (associative) brain structure in which inputs from various higher sensory areas and those from memory- and motivation-related areas converge, and whose outputs are routed to premotor and motor structures such as the arcopallium and the basal ganglia^10^,^44^. It is unlikely that cue-evoked NCL activity forms an essential part of basic visual processing. First, visual discrimination capacity is not impaired by either permanent lesion or transient inactivation of NCL^14^,^24^,^45^. Second, in working memory tasks, NCL-neurons are only weakly active during the actual stimulus presentation but increase their activity during the delay phase after stimulus offset in which the cue has to be kept in working memory^27^,^28^. Third, NCL neurons encode upcoming behavioral choices rather than current stimulus input during perceptual decision making^21^. Fourth, a recent electrophysiological study has demonstrated that NCL neurons during cue presentation extract the numerosity of the displayed items while disregarding their shape or geometrical arrangement^46^. Instead, we propose that the stimulus-related modulation we observed rather serves a ‘permissive’ or ‘informative’ role, in the sense that, to use visual stimuli to guide behavior, the NCL must receive visual information about the external world to link this information to actions and action outcomes.

**Figure 7:**
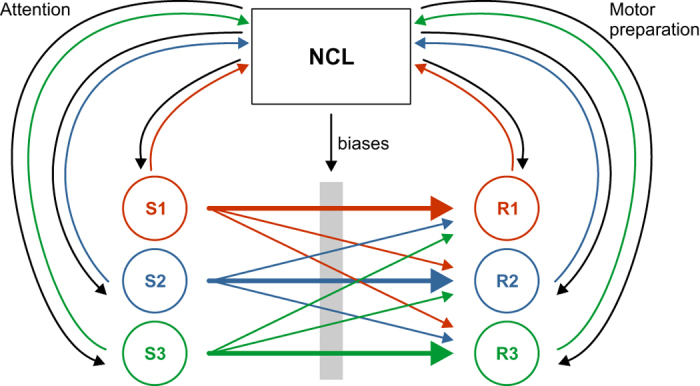
Schematic of NCL’s proposed function. Diagram inspired by Miller and Cohen^47^. S1–S3 denote environmental stimuli, R1–R3 behavioral responses. The NCL receives sensory information from higher sensory areas (inbound arrows from S1–S3) and in turn modulates sensory processing (outbound arrows to S1–S3; ‘attention’). Similarly, NCL projects to downstream motor centers and in turn receives afferent information from these centers (inbound and outbound arrows from and to NCL from R1–R3). Each stimulus has a strong connection to one of the responses (bold horizontal arrows). In simple situations, NCL does not need to interfere between ongoing stimulus-response chains (arrows from S1–S3 to R1–R3). In case of conflict, NCL biases S-R connections. See Discussion for further details.

Miller and Cohen^47^ likened the PFC to a switch operator in a system of railroad tracks. In this analogy, trains (activity e.g. carrying sensory information) must be routed to their proper destination (e.g. a behavioral response). PFC steps in when multiple trains are to be coordinated and re-routed to different destinations. In this view, the PFC is constantly fed sensory- and motor-related information to monitor the environment in relation to ongoing behavior but does only intervene when currently executed behaviors have to be interrupted, and other actions should be pursued. Taking the switch operator analogy to NCL, stimulus-related firing could simply be a signature of visual information received from higher visual areas such as the entopallium^48^. Since our paradigm, for the most part, does not require pigeons to handle ambiguous situations, NCL may not become engaged in the sense that no modulation of processing in upstream sensory or downstream motor areas or in their mutual connections is required (Fig. 7). However, some simple change to our sign-tracking paradigm, such as requiring the animals to choose between the differently valued visual cues^19^, may be enough to engage NCL circuitry to perform value-based comparisons.

If not in NCL, where might value signals be found in the avian brain during sign-tracking? In the mammalian brain, a wide range of structures have been implicated in the coding of reward, perhaps most notably the basal ganglia and dopaminergic brain stem nuclei^49^. Substantially less is known regarding reward processing in the avian brain, but several studies have demonstrated reward coding at the level of the basal ganglia in domestic chicks; for example, Izawa and colleagues^50^ found that neurons in chick ventral striatum modulated their firing rates as a function of temporal reward proximity as well as reward magnitude in an operant color-discrimination task (see ref. 51 for similar results). Another recent study employing Bengalese finches showed that neurons in Area X, a striatal nucleus of the avian song system, are modulated by food reward^52^. This study is of particular interest because their operant task bears some similarity to our sign-tracking paradigm (finches had to peck to a visual cue for reward). Moreover, neural activity in the pigeon entopallium^48^ and visual wulst^53^ was found to be related to reward-predicting properties of visual cues in operant discrimination tasks. Together, these studies suggest that reward processing may be rather widespread in the avian brain, as is the case for the primate brain^54^.

To conclude, response properties of NCL neurons are determined both by stimulus properties as well as behavioral responses in a highly context-dependent manner. A fruitful direction for future research may involve coupling stimuli with distinct but well-controlled visual properties to not a single, but several well-defined actions with specific outcomes, along with contextual changes requiring behavioral flexibility^55^,^56^,^57^; such novel paradigms are needed to break down the variability in NCL neural responses into its constituent elements and refine our understanding of the mechanisms by which this brain structure exerts executive control^58^.

## Methods

### Animals

Five homing pigeons (Columba livia *forma domestica*) served as subjects. Birds were housed individually in a colony room kept on a 12/12h light/dark cycle (lights on at 8 am). Food access was restricted to experimental sessions and weekends, with the birds being constantly kept above 85% of their free-feeding weight. Water was available *ad libitum*. Subjects were kept and treated according to the German guidelines for the care and use of animals. The experiment was approved by a national ethics committee of the State of North Rhine-Westphalia, Germany.

### Apparatus

Testing was conducted in a custom-built operant chamber measuring 33 by 35 by 36 cm (width by depth by height) and illuminated by a light bulb set into the side wall. The chamber was surrounded by a sound-attenuating shell. White noise was played at all times to mask extraneous sounds. Conditioned responses (key pecks) onto a translucent pecking key set into an opaque back well were registered by electronic switches. The pecking key measured 5 cm by 5 cm and was located 25 cm above the floor. The force of individual key pecks was registered using a custom-built piezoelectric sensor attached to the pecking key. A flat-screen monitor mounted behind the wall was used to display visual stimuli (cues). Valid responses (i.e. those which activated the switches) were acknowledged by a feedback click. Food reinforcement following stimulus presentation was provided by a food-hopper located below the response key which controlled access to a grain reservoir. During feeding, a feeder light just above the reservoir was activated.

### Behavioral Paradigm

Subjects were trained on a Pavlovian sign-tracking paradigm, in which distinct visual stimuli predicted rewards of differing magnitude, delivered after a variable delay. Figure 1A illustrates a single trial and an example stimulus set. Following an intertrial interval of 8 s, an orange initialization stimulus was displayed on the response key. After the animal pecked once at the key, a fixed interval (FI) schedule of 2 s commenced, so that the first pecking response after 2 elapsed seconds initiated the trial. Failure to respond within 2 s after the FI had elapsed aborted the trial and was marked as an initialization omission. Correct initialization was followed by presentation of one of five distinct stimuli on the response key for a fixed time of 5 s, which was succeeded by the stimulus-specific outcome, irrespective of the subject’s behavior. The CS- was followed by 2 s of mild punishment (playing an 80 Hz sawtooth wave sound and turning off the house lights), whereas the other four stimuli predicted reward at a unique combination of magnitude (i.e. time of access to the grain reservoir) and time until delivery. Magnitude could be small (1–1.5 s access to food, denoted as “m”) or large (5–6 s access to food, “M”); similarly, delay to reward could be short (1 s of delay, denoted as “d”) or long (5–6 s of delay, “D”). Reward parameters were adjusted for individual subjects within the described range to ensure a stable differentiation of stimuli for all subjects. A full stimulus set thus encompassed five stimuli: CS-, md, Md, mD, and MD.

The images representing these conditions were selected from a larger pool, so that no two animals associated the same image with a given condition. A sample stimulus set and an illustration of their associated reward properties is shown in the right half of Fig. 1A. After a 5 second presentation time, the cue was extinguished and the feeding light was turned on for the stimulus-specific delay to reward. Reward was then delivered with a reward probability of 50% for all stimuli. In case of reward, feeding light and feeder were activated for the time specified by magnitude; otherwise, the feeding light was lit for the same time. Each session contained 200 trials (40 trials per stimulus), and birds were tested five days a week.

### Behavior Analysis

During stimulus presentation, all subjects responded copiously as previously described for sign-tracking procedures^1^. We recorded pecking responses to all stimuli during the presentation time of 5 s, interpreting response rate as an indicator of subjective value^30^. To ensure reliable differentiation of stimuli, we calculated stimulus discriminability as the area under the receiver-operating characteristic curve (AUROC) for distributions of response counts to all possible stimulus pairs, and excluded all behavioral sessions in which the pairwise discriminability for any value-predicting stimuli was below 0.5. Sessions in which value-predicting stimuli did not receive at least 25 pecking responses were also excluded because a low number of key pecks precluded reliable estimation of peri-peck time histograms (see below).

Aside from response frequency, we also measured the force of pecking responses to assess whether stimuli of higher value might elicit more forceful key pecks. Pecking force was recorded via a piezoelectric transducer attached to the response key. We quantified the force of individual key pecks by rectifying and summating the vibration-induced voltage trace in a 100-ms window following registered key pecks. The measured signals were not calibrated with actual force measurements; results are thus presented in arbitrary units. We calculated stimulus discriminability by the force of responses using the AUROC characteristic, identical to what was done for response frequency.

### Surgical Procedures

After stimulus-specific pecking rates had stabilized, pigeons were implanted unilaterally in the NCL (AP +6.5−7.0 mm, ML +/− 7.5 mm) with custom-built microdrives (modified from^59^), allowing for the linear advancement of fifteen 25 μm formvar-coated nichrome wires and one 75 μm nichrome wire for differential referencing. Animals were anesthetized with isoflurane with additional regular administration of the painkiller butorphanol (0.1 ml every 2 hours). Following fixation in a stereotactic apparatus, the scalp was cut and retracted to expose the skull. Eight to ten miniature stainless steel screws were driven into the skull for subsequent implant fixation. Craniotomies were performed at the indicated positions, the dura was removed and the electrodes were lowered slowly to their final positions. A layer of vaseline was applied to the brain surface and dental acrylic was used to fix the electrodes to the skull. Animals were treated with the painkiller carprofen (concentration: 10 mg/ml, dosage: 1 mg per ml per 100 g of body weight) for three days following surgery and allowed to recover for two weeks before recommencing training.

### Electrophysiological Recordings and Data Analysis

Electrodes were advanced by at least half a revolution of the drive screw (125 μm) one hour before each recording session. Neural activity was recorded using the AlphaLab Stimulation and Recording System (Alpha Omega, Nazareth Illit, Israel). Signals were amplified 400-fold with a sampling rate of 22,321 Hz and stored in the AlphaLab file format. Offline analysis was performed in Spike2 Version 7 (Cambridge Electronic Design, Cambridge, UK), using custom-written MATLAB code. Signals were digitally band-pass filtered from 500 to 5000 Hz, putative spike events were extracted by amplitude thresholds and sorted using principal component analysis and correlation clustering to yield single-unit data. There was no preselection of neurons for any particular properties.

Since subject behavior was heterogeneous across stimuli (recall that a stable differentiation in response rate was a prerequisite for further analysis), we could not compare neuronal activity across the entire sample phase without confounding subjects’ valuation and motor output of the animal. Therefore, we controlled for differential motor output (rate of key pecking) by focusing on neural responses in the temporal vicinity of key pecks delivered onto a given stimulus. We analyzed neuronal activity within ±100 ms of registered pecking responses to construct peri-peck time histograms (PPTHs); for visualization purposes, these were convolved with a Gaussian kernel with 25 ms as standard deviation. Importantly, all analyses were conducted on raw spike counts. To avoid double inclusion of individual spikes, pecks occurring within 100 ms after the previous peck were eliminated.

We used the non-parametric Kruskal-Wallis procedure to test whether mean spike counts were significantly different (p < 0.05) across stimuli (identifying “stimulus-modulated” neurons). Furthermore, we correlated pecking responses emitted onto a given reward-predicting stimulus with the degree of neuronal modulation using Kendall’s rank correlation coefficient tau. For statistical evaluation on a population level, we compared the distribution of tau values against a shuffled distribution using the Chi-Square goodness-of-fit test. Shuffled distributions were generated by randomly allocating spike count distributions to distinct stimuli, averaging over 1,000 iterations. All analyses were performed in Matlab Version R2012a (The Mathworks, Natick, MA).

### Histology

Upon completion of the experiment, birds were deeply anesthetized with equithesin (4.5–5.5 ml/kg body weight) and transcardially perfused with 0.9% saline at 40 °C, followed by 4% formaldehyde. Prior to anesthesia, 0.1 ml heparin was injected intramuscularly to prevent blood coagulation. Brains were embedded in gelatin before sectioning at 40 μm and subsequent staining with cresyl violet. Electrode positions were reconstructed by microscopic identification of the deepest and/or widest electrode track using light microscopic observation in reference to the stereotaxic atlas of the pigeon brain^60^.

## Additional Information

**How to cite this article**: Kasties, N. *et al.* Neurons in the pigeon caudolateral nidopallium differentiate Pavlovian conditioned stimuli but not their associated reward value in a sign-tracking paradigm. *Sci. Rep.* **6**, 35469; doi: 10.1038/srep35469 (2016).
